# What Factors Influence Canadian Nurse Practitioners’ Willingness to Act as Assessors and Providers for Medical Assistance in Dying (MAID)?

**DOI:** 10.1177/23333936251390481

**Published:** 2025-11-05

**Authors:** Zachary Mokosak, Barbara Pesut, Sally Thorne

**Affiliations:** 1 University of British Columbia, Vancouver, BC, Canada; 2 University of British Columbia, Kelowna, BC, Canada

**Keywords:** assisted dying, voluntary euthanasia, nurse practitioner, end-of-life, advanced practice, suffering, death and dying, Canada

## Abstract

In passing legislation in 2016 to allow medical assistance in dying (MAID), Canada became the world’s first jurisdiction to allow nurse practitioners (NPs) to act as MAID assessors and providers. Health Canada’s annual report shows that the demand for MAID in Canada increases each year, as does the proportion of MAID cases that NPs provide. The purpose of this study was to better understand factors that motivate or deter nurse practitioners from becoming MAID assessors and providers. The study design was a secondary analysis of a large qualitative dataset guided by interpretive description methodology. Primary data collection took place from 2018 to 2023 via semi-structured interviews with nurses and NPs. Secondary analysis of transcripts of all of the NPs interviewed for the primary study allowed for identifying significant motivational and deterring themes in their accounts. The analysis yielded two categories of motivating factors (philosophical perspectives; experiences with death and dying) and three deterring factors (moral complexity; health system barriers; professional and social considerations), and further generated insights around supports and practices that make NP MAID work viable. As the first study that explicitly sought to understand what explains Canadian NPs’ willingness to participate in MAID, these findings fill a gap in the available knowledge.

## Background to MAID in the Canadian Context

Medical Assistance in Dying (MAID) was legalized in Canada in 2016, following a lengthy series of public debates and court challenges with respect to the rights of persons who have a grievous and irremediable medical condition causing enduring and intolerable suffering and who wish assistance to end their life (Library of Parliament, 2016). The initial and subsequent legislation pertaining to MAID for those without a reasonably foreseeable natural death in 2021 (C-7; Library of Parliament, 2021) included robust safeguards to prevent error and abuse in its provision. While the legislation recognizes the right of conscientious objection by individual health care providers, it also asserts that those who wish to access MAID and are found eligible under explicitly articulated criteria should be able to do so. In this manner, it expects Canadian health care systems to create access pathways for persons seeking MAID to be assessed for eligibility and, if found eligible by a minimum of two qualified assessors, to be provided with MAID.

From the outset, the Canadian legislation recognized nurse practitioners (NPs) in addition to physicians as independent eligible MAID assessors and providers, becoming the first country to do so (Pesut et al., 2019). In Canada, NPs are a separately regulated category of nurses whose advanced education and experience beyond the level of registered nurse entitles them to autonomously diagnose and treat illnesses, order and interpret tests, prescribe medications and perform many medical procedures (Canadian Institute of Health Information, 2025; Canadian Nurses Association, 2024). Despite some variations between provinces and territories in the timing of NP implementation and their role and scope of practice, NPs now function across the country in an advanced, independent nursing role. The decision to include NPs within the MAID legislation was not a foregone conclusion. In the early consultations and analysis that led to the final legislative bill on MAID, the practice was referenced as “Physician-Assisted Dying” (Wilson-Raybould, 2016). However, advocacy by numerous witnesses and briefs during the information gathering process (e.g., Philpott, 2016; Special Joint Committee on Physician-Assisted Dying, 2016; Sutherland Boal & Roussel, 2016), and the recognition that NPs were responsible for an increasing proportion of the country’s primary care in particular (Canadian Institute of Health Information, 2025), led to their inclusion in the final legislation.

As of Health Canada’s (2024) most recent annual report, NPs now represent 5.5% of the country’s 2,200 unique MAID providers (p. 9). With the expanding role of NPs in the health care system and the increasing share of MAID cases being provided by NPs, NPs have become an integral part of MAID-related care in Canada. Despite this, little is known about why NPs elect to become MAID assessors and providers. Since the legalization of MAID in 2016, research has been undertaken to understand the perceptions, experiences, and motivations of Canadian healthcare providers involved in MAID; however, these studies have tended to focus on physicians or nurses as a broad group, rather than NPs specifically. Although NPs share a foundational knowledge and approach to health care with all nurses, they also hold many of the same privileges as do physicians. As one of those privileges is the opportunity to serve as bona fide MAID assessors and providers, it seems important that we understand why individual NPs may choose or not choose to provide these services. As Canada is the first jurisdiction to allow NPs to provide full MAID services, and limited NP involvement is now legal in parts of Australia (Nicol, 2021) and under discussion toward this end in several U.S. states (Harrawood, 2024; Singer et al., 2022), this question represents important new territory. Thus, the purpose of this study was to identify factors that motivate or deter nurse practitioners’ willingness to take part in MAID as assessors and providers.

## What Prior Research Tells Us

A modest body of published research (14 papers between 2016 and 2024) provides some clues as to what might motivate NPs to participate or not participate in MAID. We know that, as the practice unfolded over the initial years, many who were initially hesitant did become involved, seeking specialized training through the Canadian Association of MAID Assessors and Providers’ MAID Curriculum (Shapiro et al., 2024). Therefore, perceptions captured in the early studies may well have evolved and changed over time.

Only three Canadian studies have focused explicitly on motivation to participate in MAID, drawing on qualitative (Brown et al., 2021a, 2021b) and quantitative (Stewart et al., 2021) explorations involving samples of physicians and NPs. From these, we know that practitioners who self-identified as non-participants in MAID have attributed that decision to exogenous factors such as (1) the health care system they work within, (2) the communities where they live, (3) their current practice context, (4) how their participation choices were visible to others, (5) the risks of participation to themselves and their family, (6) time factors, (7) the impact of participation on the patient’s family, and (8) patient–HCP relationship, and contextual factors (Brown et al., 2021a) and endogenous factors such as (1) previous personal and professional experiences, (2) comfort with death, (3) conceptualization of duty, (4) preferred EOL care approaches, (5) faith or spiritual beliefs, (6) self-accountability, (7) consideration of emotional labor, and (8) future emotional impact (Brown et al., 2021b). A third study, by Stewart et al. (2021) used a quantitative survey approach to probe motivators and deterrents related to practitioners’ desire to take part in MAID. Practitioners identified various “protective factors,” such as (1) providing compassionate/humane care/peaceful death, (2) relief of patient suffering, (3) enhancing patient choice and autonomy, (4) gratitude of patients, and (5) emotionally rewarding work, such as the honor/privilege in assisting, as well as “stressors” such as (1) future decisions about mental disorders as a sole reason for MAID, (2) decisions about mature minors in the future, (3) extra clinical workload, (4) patient’s family conflict over MAID, and (5) determination of capacity or fluctuating capacity (Stewart et al., 2021). Clearly what we know about such decisions is that they are complex and multi-faceted and, in many cases, amenable to change over time.

A number of studies have addressed the motivation question more indirectly by probing health care workers perceptions of MAID and their experiences providing such care. In one of the earliest studies, Beuthin et al. (2018) interviewed registered nurses and one NP during the first 6 months after MAID legalization to understand their experiences with providing MAID-related care or declining to participate in such care. These authors identified the desire to provide holistic care, advocate for patient choice, and help a patient achieve a “good death” as key factors. They also recognized that individual beliefs and an understanding of one’s own emotions was requisite to the practice of this care, such as responding to MAID requests, starting intravenous lines, and debriefing with families (Beuthin et al., 2018). In a secondary analysis of the same data set, Bruce and Beuthin (2020) focused on how nurses’ overall experiences of suffering were influenced by MAID, finding that nurses’ pre-existing experiences with death and suffering influenced their perceptions of dying in the context of MAID, that MAID afforded peace to those who are suffering, which brought feelings of gratitude, and that many nurses also felt residual discomfort after a MAID provision, confronting deep and abiding questions, including worry about the potential for desensitization (Bruce & Beuthin, 2020). As the legislative context has evolved over time, various researchers have focused attention on MAID assessor and provider perceptions in relation to many of the complex conditions under which MAID might be considered. In this manner, Variath et al. (2022) explored the waiver of final consent that was introduced into the legislation in 2021 for patients concerned about losing capacity, and Close et al. (2023) and Wiebe and Kelly (2023) studied the transition into MAID where reasonably foreseeable natural death was no longer a requisite eligibility criterion.

## Primary Research for this Secondary Analysis

Over this same period in time, our team has published a series of research reports deriving from interviews with nurses and NPs across Canada with respect to their perceptions of and experiences with MAID care. An initial 2018 to 2019 interview study with 32 RNs and 13 NPs led to three reports. In the first, nurses reported on the organizational contexts within which their MAID work was undertaken, including the impact of organizational leadership at both the executive-level and the program-level and the limited availability of policy and procedures to support their practice. They also commented on unit culture, including the importance of teams and teamwork, in their efforts to ensure a patient-centered approach within their current health system context (Pesut, Thorne, Schiller, et al., 2020). The second, published the same year, explored how nurses and NPs approached MAID care and developed a sense of what they considered to be optimal practice within the complex context of the emerging and evolving regulatory standards and practice supports. They emphasized the manner in which MAID availability had created new considerations and responsibilities as they engaged in conversations pertaining to end-of-life options with their patients (Pesut, Thorne, Schiller, Greig, Roussel, & Tishelman, 2020). The third probed nurses’ moral journeys in the context of MAID, including considerations that influenced their willingness to participate in MAID. These included social influences, professional experiences, and their proximity to MAID care itself. Nurses also grappled with their emotional reactions and explored potential reasons for such responses, attempting to make sense of their own moral compass with respect to MAID, and reflected on philosophical concepts such as patient choice, duty, and the afterlife (Pesut, Thorne, Storch, Chambaere, et al., 2020).

A subsequent program of study by our team expanded the initial data set into a longitudinal approach to interviewing nurses and NPs over time, thus building on the initial interviews and recruiting additional nurses and NPs through to 2023. The first analysis focused on their apprehensions with the implementation of an expanded eligibility for more complex populations as well as supporting patient choice and managing what were surfacing as practical logistics arising from MAID work complexities, increased workload, and health system integration (Pesut, Thorne et al., 2021). The second focused on nurses’ perspectives on suffering, including the existential crisis associated with the feeling of slowly losing one’s self within the experience of illness and the recognition of how little curative approaches can do to help these types of suffering. Their accounts also addressed the specific types of suffering that often lead to MAID, such as the fear of the unknown, not being able to predict the course of dying, and losing one’s dignity at the end of life. They further reported insights into the unique types of suffering which MAID can provoke, including difficulties with making the decision, the stress of being found ineligible, and the anxieties surrounding the final preparations (Pesut, Wright et al., 2021). A third analysis from this data set described the complexities of the evolution of MAID care in Canada from a nursing perspective. Nurses and NPs described how the option of MAID had introduced additional intricacies around advanced care planning, as not all patients were aware of their options, requiring additional challenges related to the timing and presentation of such an option. The waiver of final consent and the expanded availability for patients who did not have a reasonably foreseeable death also created further logistical and ethical considerations associated with their care (Pesut et al., 2024).

## Gap in the Literature

While the studies identified in this literature review help elucidate some details related to the research question of NP motivators, none answered this question directly. All of these studies included NPs as participants, but the sample cohorts mixed physicians and NPs or NPs and nurses with little disaggregation of the NP experience in the study reports (the exception being Pesut, Thorne, Schiller, Greig, Roussel, & Tishelman, 2020). Most did not focus on motivators or detractors, but rather provided hints about them through the lens of perceptions and experiences. While perceptions and experiences can be helpful for identifying factors and conditions that motivate or deter, an interpretive leap is necessary to get from one to the other. Of the three studies that did focus on factors influencing motivation, two focused on non-participants solely, with minimal differentiation between physician and NP perspectives (Brown et al., 2021a, 2021b). The quantitative study did indeed produce an extensive list of stressors and protective factors for provider involvement with MAID (Stewart et al., 2021); however, the preferences of NPs were not disaggregated from the physicians and the four-point Likert survey methodology provides little opportunity for probing depth and nuance (Bishop & Herron, 2015). More recent surveys of NPs’ views toward assisted dying are limited to NPs in jurisdictions in which it is not yet legal (Singer et al., 2022). The literature reviewed indicates a substantial amount of research has been undertaken to elucidate health care provider’s perceptions, experiences, and motivations related to MAID. However, the specific question – “what factors influence NPs willingness to take part in MAID as assessors and providers?” – remained unclarified. Thus, a focused secondary analysis of the full data set available to the team seemed an appropriate way to further study this issue.

## Secondary Analysis Methodology

The body of interview data available to us represents a significant proportion of that which has been gathered with respect to NPs’ experience over this period of time in Canada. Furthermore, in their signed consent to participate, all study participants had provided informed consent for their data to be used in subsequent studies, and an additional ethical approval was granted for this secondary analysis (University of British Columbia Behavioral Research Ethics Board #H23-03372).

Secondary analysis in the context of qualitative health research is the additional analysis of an extant body of data oriented by a new question that was not a specific line of inquiry in the initial analyses but is appropriately aligned with the original study focus and data set (Heaton, 2004; Thorne, 2013). As was the case for the primary studies, the secondary analysis used Interpretive Description methodology (Thorne, 2016) to guide data analytic processes. Explicitly developed for studies in the applied and practice fields such as health care, Interpretive Description asks what can be learned from a body of systematically constructed data, encouraging critical reflection informed by the disciplinary epistemology that led to the research question being identified, and seeks to generate new insights relevant to the practice field rather than theory building per se.

For the primary studies from which the data set for the secondary analysis was obtained, study participants were recruited in the period 2018 through 2023 using purposive and snowball sampling from all jurisdictions across Canada, except Quebec (unique provincial laws) and the Territories (low case numbers; Health Canada, 2024). Over the course of the research, study participants were interviewed 1 to 3 times, in a semi-structured manner according to an interview guide, via telephone call or Zoom. Interviews were audio recorded; once checked for accuracy, transcribed records were anonymized.

All interviews from NP participants (42 interviews with 28 different NPs) were harvested from the primary data set developed by the second and third authors (BP & ST) both of whom are RNs working closely with MAID teams but not involved directly in assessment or provision. 14 of these interviews occurred in 2018 to 2019, 18 in 2020 to 2021, 10 in 2022 to 2023 (see Supplemental Information 1). Before access was provided for secondary analysis, the transcripts were further reviewed and redacted to remove any indirectly identifiable information (e.g., province, health authority, other affiliations) to safeguard against any reidentification of study participants, given that the first author of the secondary analysis (ZM) has had experience with MAID-related policy and oversight as an administrator and may have had prior contact with participants.

Data familiarization was established through repeated readings of the transcripts, using critically reflective notes so that the data could be better appreciated in its entirety and to avoid fixation or superficial coding or categorizing before justifiable connections began to take form inductively (Thorne, 2016). This practice was maintained throughout the analytical cycle to produce an auditable line of reasoning (Thorne, 2016). Given that the original studies focused on several different questions beyond that of NP motivations, this secondary analysis focused specifically on sections of the interviews that provided insight into NPs’ willingness to act as assessors or providers for MAID. We developed a set of initial codes based on those themes then interrogated and recategorized or combined them through ongoing analysis until an organizing structure was created through which to present the findings in a manner that answered the research question. Credibility measures consistent with interpretive description methodology (e.g., epistemological integrity, representative credibility, analytic logic, interpretive authority, moral defensibility) were used throughout, and to further justify the veracity of the final thematic report, quotes from the interview transcripts were deployed to support the interpretive assertions (Thorne, 2016).

## Findings

We identified two general categories of motivating factors (philosophical perspectives, and experiences with death and dying) and three general deterring factors (moral complexity, health system barriers, and professional and social considerations). Considering these together, we came to understand the professional and personal supports these NPs require in order to engage in this complex work.

### Motivating Factors

Motivating factors (“motivators”) involved reasons NPs explicitly cited for being involved in MAID as an assessor or provider, or explanations that could be reasonably associated with such involvement, such as feeling privileged or grateful with respect to these practices. Some were factors that spurred an NP’s initial interest to act as an assessor or provider, and others explained their continued interest in this work.

#### Philosophical Perspectives

Most of the NPs interviewed within the study endorsed pre-existing perspectives about humanity and healthcare that strengthened their resolve to act as an assessor or provider. These included beliefs about individual rights and patient-centered care, as well as perspectives on how and where MAID services should be provided within the health system.

A key perspective in their accounts had to do with the importance of patient autonomy, including a patient’s right to choose what care options they prefer and under what circumstances they would like to die. As one explained “*Your job is to help people die however they want to die*” (NP18). In this context, ensuring that patients retained control at the end of life was central, even when those choices might be somewhat uncomfortable. One NP explained:

It’s an interesting thing because, on a personal level, I’m not for MAID. Being a long time older palliative care person, I was brought up in a sense that we don’t hasten death. It’s part of the definition of palliative care. But after much reflection and looking at what patient needs are, I sort of decided that it wasn’t about what I believed. It was about what the patient needed and felt they needed (NP50).

For many, a primary motivation for engaging with this practice had to do with ensuring that patients in their region or program area had access to the care for which they were eligible. “*I felt like there were cases where my patients wished to have this service and I felt it was. . . I don’t want to say a duty but I felt it would bring them comfort to walk through this journey with someone that they knew*” (NP94).

A second philosophical perspective that influenced their decision in this practice was their sense of an inherent consistency between palliative and MAID care as part of end-of-life care. Those who had provided palliative care in long term care or hospice for many years had dealt with death and dying on a regular basis. Others providing care in community typically got involved with end-of-life care only when their patients reached that stage. However, all agreed that palliative care and MAID should be available for patients at the end of life. As one NP explained, “*I always say to them. . . if I’m going to be working with you for your MAID request, let’s try and make sure your symptoms are as well managed as possible. I’d like to walk with you whatever path you choose”* (NP84). Another explained why palliative care providers can become highly skilled MAID providers. “*They can really show patients that, you know, we can do a lot of things, in addition to having that as an end-of-life option*” (NP50). Although not all NPs identified their MAID practice as deriving from palliative care expertise, a unifying theme underpinning these perspectives was that patients should not have to choose one over the other.

A third prominent philosophical perspective across the NPs interviewed was a sense that this practice was a clear fit with nursing’s underlying core values and expertise. Several expressed gratitude that the legislation included NPs as well as physicians in this work, as they felt that NPs were needed to support the public demand for MAID, especially in under resourced areas. “*You know, why do NPs exist? Because of a gap in the system”* (NP18). As MAID administration is primarily via intravenous injection in Canada, their nursing skillsets meant that they were proficient in starting IVs without the need to bring in an extra RN provider, “*So I don’t have limits there*” (NP25). Others noted that the highly relational skills fundamental to their nursing background were a major asset to MAID practice. *“You can build up a rapport with them and be therapeutic. We’ve had years and years of practice doing that, making somebody feel comfortable*” (NP08). Yet another aspect of this fit with the essence of nursing was its inherently holistic approach. “*Nurse practitioners are the ideal practitioners for doing Medical Assistance in Dying because our foundational learning is on caring. . . There is no curing; MAID is all about caring*” (NP30).

#### Experiences with Death and Dying

Prior experiences with complex dying patients they had cared for and harrowing deaths they had witnessed were often instrumental in their decision making with respect to becoming MAID assessors and providers. As one recalled, “*I’ve also had other palliative experiences when I worked as a young [RN] grad where I watched patients suffer in terrible pain. And no matter how much medication we had available and gave to that client, they still went out screaming and suffering*” (NP26). Another explained, “*I think my work as a shock trauma nurse traumatized me. . . You know, I still think about the things I saw, the way people died*” (NP88). Those who referenced such difficult prior nursing experiences inevitably described feelings of helplessness and memories which weighed heavily on them. As one commented, “*I think medicine and health care in general is really finding we don’t like to say that there’s nothing more we can do*” (NP08).

Given these experiences, many welcomed the inception of MAID as an option to relieve the kind of undue suffering they had seen in their practice, and consequently found the nature of MAID work to be professionally fulfilling. As one said, “*Honestly, I’m more driven to help people address their suffering than I’ve ever been before, by experiencing and knowing that this person got an end to that suffering and I was able to help with that was very uplifting, despite how hard it was*” (NP79). Others referenced a sense of awe they felt in working with patients who had elected this option. “*They’re making a very conscious decision that they are going to end their lives and, you know, there’s a courage of that that, you know, is very humbling*” (NP2). Many also acknowledged that MAID was giving patients back a sense of control over the conditions of their death, and the opportunity to reflect on the legacy they were leaving behind. “*Well, to me these are legacy conversations and this is a legacy issue, just the time we’re spending, it’s very profound*” (NP1). Others explicitly referenced the privilege in being able to help family members with the passing of a loved one. “*And then, the responses from the family members. You know, I can’t believe how, you know, peaceful that was. They were so comfortable, you were so kind, this is just the way they would’ve wanted to die. You know, those kinds of things are. . . they stay with you*” (NP30).

### Deterring Factors

Deterring factors (“deterrents”) involved issues NPs explicitly acknowledged as reasons for not taking part in MAID, or could be reasonably associated with their concerns about involvement, such as ethical or moral concerns.

#### Moral Complexity

As a novel service in Canada, MAID presented NPs with a dense moral and ethical landscape. These included MAID-related cases they felt were difficult to rationalize and reconcile, such as in the context of perceived health system failures, patient and family grief, or blurred ethical lines. Although feelings about such moral complexity were often difficult to articulate directly, they were often shared in the context of patient stories, and it was apparent that these feelings could be profound.

For many of these NPs, confronting some degree of moral distress was an inherent part of their MAID care journey. For example, some referenced the fundamental tenet in health care of “do no harm.” “*You know, it’s taking everything that we have normally done and turning it upside down*” (NP13). Others struggled to reconcile the dissonance between their support for patient autonomy and their personal disagreement with some of the decisions that patients make in reality. “*I found myself kind of thinking, ‘You have lots to live for.’ But, you know, then, I had to stop and kind of say, ‘Wait you’re being. . . this isn’t about you’*” (NP49). In other accounts, NPs struggled with their conceptions of the divine and existential judgment. One NP, who was willing to serve as an assessor but uncomfortable taking on the provider role, explained “*I don’t really want to play God*” (NP13). Another NP recounted a poignant conversation with a patient in the process of preparing for MAID. “*[She] said to me. . . . ‘Just before you do this, ‘I’m not sure how I’m going to be judged. . . You know, when I’m in front of our Maker, when I’m at the pearly gates, what is going to happen because of this?’ And I said to her, as comically as I could, I said. . . “You? What about me?’”* (NP18).

MAID was described by many NPs as an intensely emotional experience. Where the patient did not have a reasonably foreseeable natural death (which came to be known as “Track 2 MAID”), the work could be especially “*emotionally and time-intense*” (NP30). Similarly, cases in which NPs had to find patients ineligible were highly distressing. “*I just couldn’t. . . didn’t feel comfortable saying yes, and it was really hard*” (NP79). While many of the NPs associated their emotional responses with particularly difficult scenarios, others described strong emotional reactions with no explainable root cause. One NP recalled their first MAID case, “*It was very emotional. . . and I went home and started a journal because of this, because I couldn’t figure out. . . Like, we debriefed, but it was still bugging me*” (NP2). Another accepted the reality that providing MAID was an inherently emotionally strenuous service “*And so, I’m. . . dealing with it now, [but] I still cry to this day.”* (NP18).

These NPs also confronted various ethical tensions associated with how MAID legislation was being applied within their current practice context. Such tensions included a perception that a colleague was trying to move a MAID provision along too quickly, or a concern that other providers were not being appropriately rigorous in their assessments. “*The danger in* that is *that people will just apply those criteria very much like a menu*” (NP50). Some worried about future conditions, should Canada’s legislation evolve to allow for MAID for persons who lacked the cognitive capacity to consent, or for mature minors, for example. Several NPs also had worries about the ethics of providing MAID for Track 2 patients. As one explained “*I’m sure there will be a lot of Track 2 cases where there is legitimate suffering. . . But some cases, I think, are shining a light on the dearth of resources to support certain conditions, lack of housing, loneliness, deplorable living conditions*” (NP84). And another expressed the moral complexity more broadly:

I think part of my whole challenge with MAID is, you know, when we guarantee. . . we have. . . you know, our government and our country has guaranteed people the right to die when we haven’t guaranteed people the right for comfort and quality of life and so I think that weighs on me quite heavily that we still don’t have universal access to good palliative care. (NP77)

#### Health System Barriers

Although NPs have bone fide legal status as MAID assessors and providers, many noted barriers in accommodating them into this practice, often associated with the relatively new status of the professional designation and the evolving nature of the practice. This was also complicated by the confusion of the early days after MAID legislation in 2016, when professional regulators and healthcare organizations tried to fill gaps in the legislation with various policies and guidance documents. The rapidity of this process and the numerous authorities involved created a situation in which there were inexplicable practice variations and unanswered questions that often left healthcare providers asking questions. For example, one recalled “*We are the only group within Canada* that is *required to have education prior to aiding MAID, and the College has tried to go on and describe what aiding means. But whenever you make a rule, then that rule makes questions*” (NP4).

Almost all of those interviewed noted challenges around compensation. Some NPs were not allowed to provide MAID during workhours, so their only option to provide MAID was on their own time; others whose MAID work would have been compensated under their salary had they been able to provide it during work hours, but went uncompensated when patients wanted MAID during evenings or weekends. “*It’s terribly frustrating and I can’t imagine that very many people would stick it out, to be quite honest, especially if you are working and have a family and finances are an issue*” (NP30). While some declined to provide services for which they were not compensated, others viewed themselves as volunteers and felt duty bound to provide it to ensure patient access. *“There were no MAID providers in the community in that time and there was a waitlist. And I felt it was the right thing to do because [they] would rather die in their home than in the hospital and I felt that it was the right thing to do to facilitate that*” (NP85). A few took it upon themselves to try and drive change in the system. “*We did a huge advocacy campaign, including restricting practice. So, we pulled back and didn’t take any new referrals*” (NP30)

Another barrier had to do with the demands of acting as a MAID assessor or provider, which could stretch their professional capacity thin, even in a relatively straight-forward case.

It’s a lot of work and you really want to be available to the patient to be able to provide what you can. But the logistics of filling 16 pieces of paper and making sure they’re all

correct. . . going to pick up the medication, and organizing with the family, organizing with the nurse. . . like, there is so much that goes into it. And that part can be so draining. (NP25)

Given the significant time commitment, even during paid workhours, some NPs felt that MAID provision was forcing them to choose between their routine patients and their MAID cases. “*I’m a palliative care nurse practitioner and that’s what I’ve chosen to do and I don’t want MAID to be something that would limit my availability for patients requiring palliative care*” (NP50). Primary care NPs working in community often felt like they were at a disadvantage. One explained, “*I can spend eight hours just trying to find the information that I need for some of the other patients. . . you’re sending fax after fax and calling and, you know, they send you to this person and that person sends you to another person*” (NP30). Another noted, “*I mean, our Track 2 assessment can take upwards of twenty hours by the time you get all the documentation*” (P85). Large, dispersed geographic regions also presented additional barriers related to travel and medication logistics. “*The farthest I’ve been to a provision is four and a half hours drive one way”* (NP89).

Finally, NPs noted that the legalization of MAID had created a novel situation for the healthcare system, insofar as healthcare providers became responsible for interpreting criminal statutes to provide a clinical service. This left MAID assessors and providers looking for legal support to understand how to appropriately apply the MAID criteria and safeguards. “*There was lots of the health care community really struggling with the legal language of the legislation*” (NP2). Because MAID was a healthcare service with criminal implications, some Canadian provinces implemented oversight bodies and systems to ensure that MAID assessors and providers apply the legislation in a rigorous manner. Many NPs understood the reason that these systems were put in place, but also conceded that they were nervous speaking to these bodies. “*The most stressful thing about doing a provision. . . is talking to the coroner’s office*” (NP30).

#### Professional and Social Considerations

NPs were fully aware that not all Canadians support the legalization of MAID, and even those who have a generally favorable view of MAID may often have deep-rooted feelings about death and dying that are difficult to reconcile with the practice. Therefore, fears of stigma and judgment related to MAID served as a complication in both their professional and personal lives. Within the healthcare community, NPs were aware of judgment from some colleagues based on their MAID involvement. One NP recounted a confrontation with staff in a faith-based hospice. “*I knew she was eligible but the hospice was doing everything they could to try to negate my decision. . . critiquing my notes saying that they were inaccurate, you know. I had people calling the [MAID Team] saying that what I was doing was not right*” (NP94). Another reported a colleague as saying, “‘*Well, you’re going to be known as ‘the lady of murder’ or something to that effect and that was really hurtful*” (NP88).

Family members of patients requesting MAID were also a common source of judgment. One NP recalled being quite shaken when a family member compared MAID providers to “*executioners*” (NP1). Another discussed the difficulties of providing MAID for patients who do not want their family members to know, because of their predictable disapproval:

You know, it makes it very awkward for everybody because you know, the family obviously they’re going to find out eventually. . .. They have every right to do that, but of course, once they’re dead, you know, that’s not where the angst goes to. It goes to the person that provided the MAID. . . ‘how dare you do that; how dare you not tell us what’s going on (NP13).

Small town dynamics could also factor into NPs’ worries. “*It’s just the culture here is very different*” (NP49). In some cases, small social circles significantly compromised privacy. “*So if the neighbor knew that someone had cancer, they might not know that they’re going to do end-of-life. But if they saw my car and someone else’s car in the driveway that day and then that person died. . . And then the rumors start”* (NP17).

### Supports Essential to MAID Practice

The accounts of motivators and detractors make it clear that, although assessing for and providing MAID can be a rich and rewarding experience, it is also a complex and exhausting form of NP practice. Given the nature of these motivators and detractors, NPs felt that the viability of MAID practice required the presence of various personal and practice supports to enable both efficient, high-quality MAID services and to maintain their personal and emotional balance.

NPs recognized that providing MAID requires extensive logistical coordination. The logistic supports provided by MAID coordination teams were highly appreciated, with one NP calling them “*invaluable*” (NP85). Flexible scheduling was also beneficial, since MAID is typically not an NP’s fulltime job. “*I’ve always held a .8 FTE, so I always have space in my calendar to take on more patients than those who work fulltime*” (NP36). Further, professional and organizational leadership support often played an important role in creating motivation and positive experiences for NPs involved with MAID:

So, my director here actually has been helpful. . . I’m going out and volunteering my time and doing MAID because of what’s happening. He said. . . ‘There’s a need in the community. People are suffering.’ He says go and do it. Your conscience is protected. In the end, what you’re doing is right.’ And so, that made me. . . that was more of a tipping point. (NP18)

NPs also valued informal supports and formed such networks with colleagues in the practice. “I have been very fortunate to be surrounded by incredible mentors, both physicians, nurse practitioners and the [MAID Teams]. And so, every step of the way, I’ve felt very prepared and very informed” (NP65). The Canadian Association for MAID Assessors & Providers was a frequently cited support. “They’re a wonderful resource, especially when you’re coming across a patient you were not quite sure what to do with or certain barriers” (NP36). The opportunity to work with and learn from mentors was profoundly influential. “To me, I think that it is essential to have someone there who is really dealing with the task. . . and it is a task when you’re administering the medication for sure but you also have someone else there who’s keeping an eye on the family” (NP1).

Even NPs who found MAID work profoundly rewarding also described scenarios of emotional exhaustion associated with its practical and ethical challenges. For many, supportive friends and family were essential to recognizing and rationalizing these feelings and concerns. “*I have great support outside of work with family and friends, so that helps significantly”* (NP105). Access to unbiased counselors was also helpful for some. “*If the person you can talk to, you know that things are confidential and they’re professional and they don’t offer any opinion. They just listen*” (NP17). Others found that speaking with *“another MAID provider who has experienced similar things and challenges*” [NP85] was much more helpful than talking to a counseling professional. Beyond these human support systems, several NPs also spoke of practices that they deliberately or opportunistically engage in to maintain emotional stability and avoid burnout. One described careful pacing as a strategy to avoid becoming overwhelmed. “W*hen that’s starting to disrupt my sleep and affect my ability to interact with my kids in a motherly way, sometimes I have to just push things back*” (NP94). Others spoke of finding peace in hobbies and other vocations “*We’re farmers and I have a real connection to being outside and being with the animals*” (NP88). Yet others created self-care practices out of everyday activities. “*I drive a lot, right, because I’m all over the region. So, usually for me, honestly, it’s just turning up the music and just thinking while I’m driving home*” (NP49).

Given appropriate supports and systems, many of these NPs described MAID practice as changing their lives for the better. “You know, I’ve had to search my own soul about my own beliefs about how I want to live my life on a personal level, as well as my practice, and clinically what I want to do with my practice. So, to me, it’s changed my career in a lot of ways” (NP2). Another explained how it provided a greater appreciation for life in general:

I guess I’ve learned a lot about myself and what’s important to me. So, it’s definitely made me reflect on my own life and what I would want my own end-of-life to be, what’s important in terms of family and quality of life. I think those are things that I thought about a lot before, just being the person I am, but being exposed to it on a very regular basis, it makes me reflect even more and really appreciate life and appreciate family and appreciate nature and love and everything in the world that we all cherish. I think seeing and being exposed to death, which, you know, ultimately MAID is a beautiful death but it is death and it’s loss, it does make me appreciate what I have. (NP25)

## Discussion

Not surprisingly, many of the factors already identified in the literature as motivators or detractors with respect to health care professional willingness to take part in MAID as assessors and providers are highly relevant to NPs as well. The kinds of endogenous and exogenous factors delineated in the research by Brown et al. (2021a, 2021b) and Stewart et al. (2021) clearly resonate with these NPs, in that they are motivated by insights derived from their professional experiences with caring for patients who are suffering and by their work with death and dying. They are further motivated by a desire to provide compassionate care, to support patients toward a peaceful death under conditions of their own choosing, and to respect patient rights and autonomy within the Canadian legislation. They also recognize the many detractors to this work that have been described in these prior studies. Community complexities, including the public profile a provider has within smaller or contained communities, poses risks that are of concern to many. Logistic challenges and system support gaps are significant issues. And they fully appreciate the moral and ethical enormity of MAID practice as an especially daunting aspect of taking on this practice.

Unlike some of the earlier literature, in which it had been assumed that individual health care providers would assume and retain either a proponent or adversarial position with respect to MAID (Berghs et al., 2005; Elmore et al., 2018; Vézina-Im et al., 2014), these NP accounts demonstrated considerable critical reflection and evolution with respect to the complex constellation of motivators and detractors with which they wrestled in the process of determining the direction of their own practices. This wrestling might be most prominent with respect to logistical and system issues for some, and deeply felt moral or ethical convictions for others. However, as their engagement with MAID and/or with patients who were considering MAID evolved, the factors that directly influenced their decisions to participate or not became increasingly complex and layered.

A unifying thread throughout their accounts was a deeply abiding respect for the patient in the full context of their medical conditions and life circumstances. Many invoked their nursing background and the core professional values it represented as their primary guide through to MAID decision making. Although they highly valued the additional diagnostic and treatment skillsets their NP designation represented, their thoughts often returned to foundational nursing knowledge about patients, about suffering, and about the primacy of compassionate care. Their willingness to continue to provide care amidst the moral complexity, lack of adequate supports in healthcare, and the potential for significant social stigma attest to this patient-focused commitment. In some U.S. jurisdictions, NPs have been found to be overrepresented among primary care practitioners in their uptake of advanced care planning in general (Harrawood, 2024). For example, in West Virginia, they complete almost twice as many advanced directives forms than do physicians and are more likely to include preferences for comfort and do-not-resuscitate orders (Constantine et al., 2021). Similarly, studies from Oregon (Hayes et al., 2017) and Vermont (Landry et al., 2020) demonstrate that NPs have a propensity among primary care practitioners toward discussions about end of life.

In recognition of the rapid expansion of nurse practitioners into MAID practice and the increasing involvement with MAID patients by nurses of all designations, the Canadian Nurses Association has explicitly drawn on MAID care as an emerging context within the core values of nursing must be applied. Its newly updated Code of Ethics for Nurses (Canadian Nurses Association, 2025) requires that all nurses reflect on their moral values and beliefs so as to anticipate when those may conflict with a person’s needs, preferences, wishes or decisions. It requires that they understand applicable legislation, regulatory standards and practice frameworks with respect to practices such as MAID, and calls on all nurses to ensure that patients are making informed decisions about such practices free from coercion and rooted in dignity, autonomy, and relational trust. Closely observing the Canadian experience, nurses and nurse practitioners in other nations are also beginning to advocate for expansions in their role with respect to involvement in voluntary assisted dying (Harrawood, 2024; Jeanneret & Prince, 2024).

While this study is limited with respect to being a secondary analysis based on interview data in which nurse practitioners were reporting on all facets of their MAID-related practice experience, it allows for confirmation of the rationale for NPs to decide to take on this role and the intellectual and philosophical processes by which they reflect on that decision. It further confirms the wider context within which they are thinking about their MAID practice and contributing to the wider Canadian discourse around MAID as it unfolds over time. On the basis of this research, it will be important to conduct further research into the possibility that NP MAID practice both conforms to the legislative ideals and also reflects a distinctive nursing angle of vision.

## Conclusion

Canadian NPs are increasingly embracing an active role in MAID practice and are making an active contribution to the public and professional dialog in this context. Their reasons for and against this participation are multiple and complex, shaped by factors that are common to those of other eligible professions but also distinctively shaped by their nursing grounding.

## Supplemental Material

sj-docx-1-gqn-10.1177_23333936251390481 – Supplemental material for What Factors Influence Canadian Nurse Practitioners’ Willingness to Act as Assessors and Providers for Medical Assistance in Dying (MAID)? Supplemental material, sj-docx-1-gqn-10.1177_23333936251390481 for What Factors Influence Canadian Nurse Practitioners’ Willingness to Act as Assessors and Providers for Medical Assistance in Dying (MAID)? by Zachary Mokosak, Barbara Pesut and Sally Thorne in Global Qualitative Nursing Research
