# Supplementary material for: What Factors Influence Canadian Nurse Practitioners’ Willingness to Act as Assessors and Providers for Medical Assistance in Dying (MAID)?
Source: Glob Qual Nurs Res. 2025 Nov 5;12:23333936251390481. doi: 10.1177/23333936251390481 (PMC12592663; doi:10.1177/23333936251390481)
Supplement: sj-docx-1-gqn-10.1177_23333936251390481 – Supplemental material for What Factors Influence Canadian Nurse Practitioners’ Willingness to Act as Assessors and Providers for Medical Assistance in Dying (MAID)? [file sj-docx-1-gqn-10.1177_23333936251390481.docx]

**Supplementary Information 1: Nurse Practitioner Demographics**

| **Variable** | **Options** | | **N** | **%** | |
| --- | --- | --- | --- | --- | --- |
| Province of Practice at 1^st^ interview | British Columbia | | 12 | 42.9 | |
|  | Ontario | | 13 | 46.4 | |
|  | Other Provinces | | 3 | 10.7 | |
| Geographic Context of Work at 1^st^ interview | Urban (population of > 10,000) | | 15 | 53.6 | |
|  | Rural (population < 10,000) | | 5 | 17.9 | |
|  | Remote | | 2 | 7.1 | |
|  | Both Urban and Rural | | 3 | 10.7 | |
|  | All | | 3 | 10.7 | |
| Work Context at 1^st^ interview | Community | | 10 | 35.7 | |
|  | Home Care | | 3 | 10.7 | |
|  | Residential or Long-Term Care | | 3 | 10.7 | |
|  | Hospital | | 2 | 7.1 | |
|  | Multiple or Other | | 10 | 35.7 | |
| Years in practice at time of 1^st^ interview | Fewer than 10 | | 6 | 21.4 | |
|  | 10 or more | | 22 | 78.6 | |
| Self-Identified Gender at 1^st^ interview | Female | | 24 | 85.7 | |
|  | Male | | 4 | 14.3 | |
| Visible Minority[^1^](#_bookmark44) | Visible minority | 2 | | | 7.1 |
|  | Not a visible minority | 26 | | | 92.9 |
| Age range at 1^st^ interview | 25-34 | 4 | | | 14.3 |
|  | 35-44 | 7 | | | 25.0 |
|  | 45-54 | 9 | | | 32.1 |
|  | 55 or older | 8 | | | 28.6 |
| Conscientious Objector to MAiD? At 1^st^ interview | Yes | 0 | | | 0 |
|  | No | 26 | | | 92.6 |
|  | Uncertain or Missing | 2 | | | 7.1 |
| Spiritual/Religious | Religious | 3 | | | 10.7 |
|  | Spiritual | 12 | | | 42.9 |
|  | Both religious and spiritual | 5 | | | 17.9 |
|  | Not religious or spiritual | 8 | | | 28.6 |
| Number of persons who have received MAID services at most recent interview | 0-4 | 5 | | | 17.9 |
|  | 5-9 | 5 | | | 17.9 |
|  | 10-14 | 3 | | | 10.7 |
|  | 15-19 | 2 | | | 7.1 |
|  | 20-24 | 2 | | | 7.1 |
|  | 25 or more | 11 | | | 39.3 |

Note: Race and Ethnicity were asked as open-ended and then categorized using Statistics Canada groups and to maintain confidentiality, further reduced to Statistics Canada visible minority and population group reference guide, Census of Population, 2021. https://www12.statcan.gc.ca/census-recensement/2021/ref/98- 500/006/98-500-x2021006-eng.cfm and Statistics Canada. Classification of visible minority. https://www23.statcan.gc.ca/imdb/p3VD.pl?Function=getVD&TVD=1323643
